# Visible Red Light Emitting Diode Photobiomodulation for Skin Fibrosis: Key Molecular Pathways

**DOI:** 10.1007/s13671-016-0141-x

**Published:** 2016-04-16

**Authors:** Andrew Mamalis, Daniel Siegel, Jared Jagdeo

**Affiliations:** Department of Dermatology, University of California at Davis, Sacramento, CA USA; Dermatology Service, Sacramento VA Medical Center, Mather, CA USA; Department of Dermatology, SUNY Downstate, Brooklyn, NY USA

**Keywords:** Skin fibrosis, LED, Visible light, Red light, Fibroblast, Low level light therapy, Photobiomodulation, Reactive oxygen species, Collagen

## Abstract

Skin fibrosis, also known as skin scarring, is an important global health problem that affects an estimated 100 million persons per year worldwide. Current therapies are associated with significant side effects and even with combination therapy, progression, and recurrence is common. Our goal is to review the available published data available on light-emitting diode-generated (LED) red light phototherapy for treatment of skin fibrosis. A search of the published literature from 1 January 2000 to present on the effects of visible red light on skin fibrosis, and related pathways was performed in January 2016. A search of PubMed and EMBASE was completed using specific keywords and MeSH terms. “Fibrosis” OR “skin fibrosis” OR “collagen” was combined with (“light emitting diode,” “LED,” “laser,” or “red light”). The articles that were original research studies investigating the use of visible red light to treat skin fibrosis or related pathways were selected for inclusion. Our systematic search returned a total of 1376 articles. Duplicate articles were removed resulting in 1189 unique articles, and 133 non-English articles were excluded. From these articles, we identified six articles related to LED effects on skin fibrosis and dermal fibroblasts. We augmented our discussion with additional in vitro data on related pathways. LED phototherapy is an emerging therapeutic modality for treatment of skin fibrosis. There is a growing body of evidence demonstrating that visible LED light, especially in the red spectrum, is capable of modulating key cellular characteristic associated with skin fibrosis. We anticipate that as the understanding of LED-RL’s biochemical mechanisms and clinical effects continue to advance, additional therapeutic targets in related pathways may emerge. We believe that the use of LED-RL, in combination with existing and new therapies, has the potential to alter the current treatment paradigm of skin fibrosis. There is a current lack of clinical trials investigating the efficacy of LED-RL to treat skin fibrosis. Randomized clinical trials are needed to demonstrate visible red light’s clinical efficacy on different types of skin fibrosis.

## Introduction

**Fig. 1:**
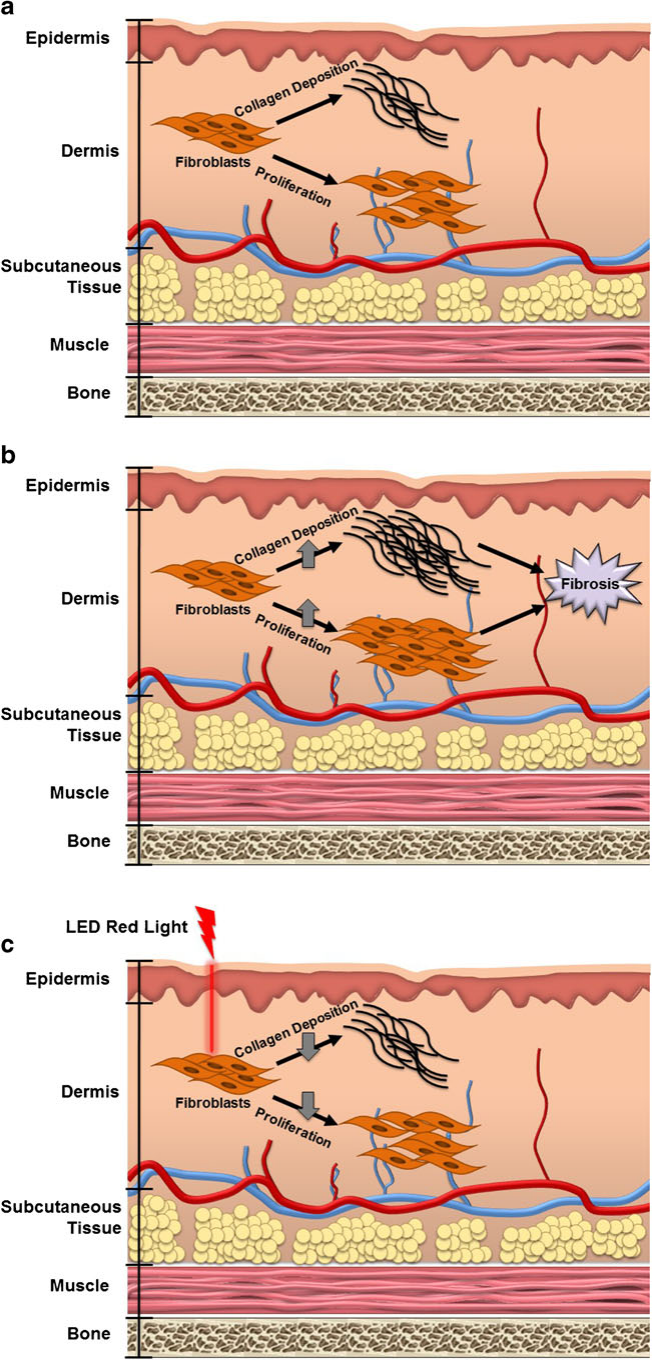
**a** Normal fibroblast function. Fibroblasts are the primary resident cell in the dermis and are the major contributor to skin fibrosis. Fibroblasts typically proliferate and produce collagen at a basal rate to maintain dermal integrity. **b** Abnormal fibroblast function increases proliferation and collagen production leading to skin fibrosis. Fibroblasts contributing to skin fibrosis have an increased proliferation rate and an increased collagen production and deposition rate. These cellular alterations are the hallmark of skin fibrosis, and thus are targets of therapeutic interest. **c** Light-emitting diode-generated red light (LED-RL) reduces fibroblast proliferation and collagen production. LED-RL alters fibroblast function leading to decreased collagen production and fibroblast proliferation. If LED-RL is capable of returning fibroblast activity to basal levels, LED-RL may be a therapeutic option for the prevention or treatment of skin fibrosis

Skin fibrosis, also known as skin scarring, is a significant international health problem with an estimated incidence of greater than 100 million persons affected per annum worldwide [1, 2]. Skin fibrosis is the key clinical characteristic of several diseases including systemic sclerosis, morphea, keloids, hypertrophic scars, chronic graft versus host disease, and gadolinium-induced nephrogenic systemic fibrosis. Skin fibrosis often results from chronic tissue injury, infection, inflammation, or immune response leading to fibroblast activation. The hallmarks of skin fibrosis are increased fibroblast proliferation, increased collagen production, increased extracellular matrix (ECM) deposition, and upregulation of pro-fibrotic signaling pathways (Fig. 1). Despite the morbidity and socioeconomic burdens associated with skin fibrosis, there are limited effective therapeutic options for skin fibrosis. Current therapies are associated with significant side effects and even with combination therapy, progression, and recurrence often occurs [3, 4].

Ultraviolet (UV) phototherapy is a non-invasive modality that has been used to treat several diseases associated with skin fibrosis including morphea, systemic sclerosis, chronic graft versus host disease, and nephrogenic systemic fibrosis [5–7]. However, UV phototherapy causes thymidine dimer DNA damage that is associated with an increased incidence of skin cancers and premature photoaging [8–10]. In addition to these safety concerns, UV phototherapy units are often prohibitively expensive for home use and require fluorescent or incandescent bulbs that limit portability. Therefore, UV phototherapy requires frequent office visits that patients often find burdensome [11, 12]. In contrast, light-emitting diode-generated red light (LED-RL) phototherapy is a safe, non-invasive, inexpensive, and portable treatment that may be combined with existing treatment modalities. Furthermore, the visible red light spectrum has superior depth of penetration, when compared to UV light, that allows it to penetrate the epidermis and reach the dermis to affect fibroblast function [13]. LED-RL is not known to cause thymidine dimer DNA damage or to be associated with an increased incidence of skin cancer [14]. However, the underlying biochemical mechanisms and clinical effects of visible light photobiomodulation of skin fibrosis are not well characterized.

The purpose of this review is to review the available evidence on LED-RL phototherapy for the treatment of skin fibrosis, with a special emphasis on the key molecular pathways involved. Herein, we also highlight several strengths and limitations of visible red light phototherapy and suggest enhancements and future directions to evaluate their clinical utility. We anticipate that as the understanding of LED-RL’s biochemical mechanisms and clinical effects continue to advance, additional therapeutic targets in related pathways may emerge. We believe that the use of LED-RL, in combination with existing and new therapies, has the potential to alter the current treatment paradigm of skin fibrosis.

## Methods

A search of the published literature from 1 January 2000 to present on the effects of visible red light on skin fibrosis and related pathways was performed in January 2016. A search of PubMed and EMBASE was done using specific keywords and MeSH terms. “Fibrosis” OR “skin fibrosis” was combined with (“light emitting diode,” “LED,” “laser,” or “red light”). The articles that were original research studies that investigated the use of visible red light to treat skin fibrosis or related pathways were selected for inclusion. Non-English articles were excluded.

## Results

**Fig. 2:**
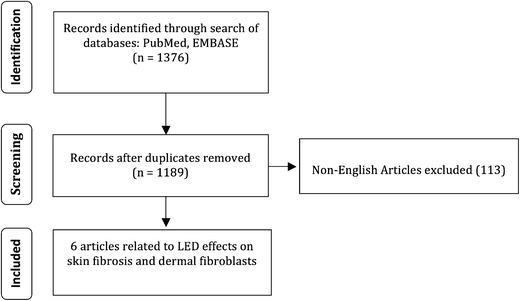
Schematic of the search strategy listing the number of articles matching inclusion or exclusion criteria

A schematic of our search strategy is outlined in Fig. 2. Our systematic search returned a total of 1376 articles. Duplicate articles were removed resulting in 1189 unique articles, and 133 non-English articles were excluded. From these articles, we identified six articles related to LED effects on skin fibrosis and dermal fibroblasts. We augmented our discussion with additional in vitro data on related pathways.

## Discussion

### Molecular Mechanism of Red Light Photobiomodulation

Although visible light makes up 44 % of the total solar energy in our environment, its effect on cellular function and physiology are not fully established [14]. There is emerging in vitro mechanistic data demonstrating red light may be an effective treatment for skin fibrosis; however, there is a paucity of evidence for red light’s clinical effects. Visible light may represent a safer therapeutic modality compared to UV light as it does not generate DNA damage associated with skin cancer [14].

**Fig. 3:**
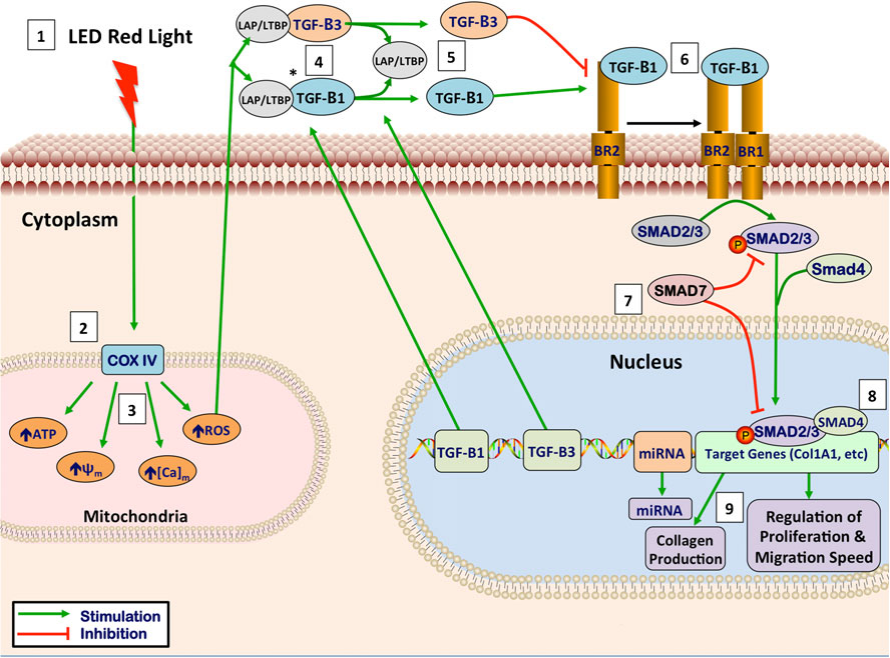
Theoretical mechanism of LED red light photobiomodulation. *1* Light has optimal tissue penetration when its wavelength is within the “optical window,” (600–1070 nm). Red light (620–750 nm) takes advantage of this penetration window [15]. *2* LED-RL stimulates the photo-acceptor copper complex in cytochrome C oxidase, stimulating the photodissociation of nitric oxide (NO), leading to upregulation of the electron transport chain [15, 16]. *3* This stimulation of the electron transport leads to the following intramitochondrial changes: increased generation of ATP and reactive oxygen species (ROS), increased intramitochondrial calcium concentration, and an increase in the mitochondrial membrane potential [16]. *4* TGF-Betas are secreted associated with latency-associated peptide (LAP). These associated latency peptides determine the activity TGF-Beta subtypes [17]. Reactive oxygen species have been shown to trigger a conformational change in LAP, thus freeing TGF-Beta 1 from its latency peptide [17–20]. It has been suggested that activation of TGF-Beta 3 may function by a similar ROS-induced release of LAP [20]. *5* TGF-B1, upon being released from its latency complex, binds to the TGF-Beta receptor II (TGF-BRII) stimulating activation [21]. TGF-B3 inhibits activation of the TGF-BRII and antagonizes TGF-B1 signaling [21]. *6* TGF-BRII then forms a heteromeric complex with TGF-BRI, causing the phosphorylation of specific serine residues [21, 22]. *7* When activated, the intracytoplasmic domain of the TGF-Beta receptor complex phosphorylates SMAD proteins. Once phosphorylated, pSMAD2/3 has the ability to migrate to the nucleus and associates with DNA-binding partners to cause changes in target gene expression [22]. SMAD7 activation functions as a negative feedback loop, inhibiting TGF-B1 signaling [23]. *8* SMAD signaling alters COL1A1 gene expression leading to changes in extracellular collagen deposition and changes in fibroblast proliferation [24]. In addition, SMAD signaling contributes to the collagen deposition and pathogenesis of skin fibrosis [25]. These pathways, and related pathways such as Akt, are believed to contribute to skin fibrosis through modulation of fibroblast proliferation and migration speed [26]. *9* TGF-B1 has been shown to increase cell proliferation at low levels; however, high levels of TGF-B1 inhibits dermal fibroblast proliferation, supporting the idea that modulation of levels of TGF-B1 may contribute to LED-RL’s modulation of proliferation [27]. In addition, the release of TGF-B1 or TGF-B3 from their LAP has been suggested as a possible mechanism behind the photobiomodulation of LED-RL [20]. Figure legend: *ψm*, mitochondrial membrane potential; *[Ca]m*, intramitochondrial calcium concentration; *ATP*, adenosine triphosphate; *ROS*, reactive oxygen species; *LAP/LTBP*, latency-associate peptide/latent transforming growth factor beta binding protein; *TGF-B1*, transforming growth factor beta-1; *TGF-B3*, transforming growth factor beta-3; *BR2*, transforming growth factor receptor II; *BR1*, transforming growth factor receptor I; *miRNA*, microRNA

A potential mechanistic pathway demonstrating the cellular effects of red light photobiomodulation in skin fibrosis is diagrammed in Fig. 3. Key downstream targets for the modulation of skin fibrosis include reducing cellular fibroblast proliferation and migration speed, inhibiting pro-fibrotic cytokines and their related pathways such as the transforming growth factor-beta (TGF-beta) pathway, and decreasing synthesis and deposition of collagen.

### Mitochondrial Signaling

The molecular mechanism behind LED-RL’s photobiomodulatory effects appears to initiate in the mitochondria [28, 29]. Red light stimulates the copper/heme-iron centers on cytochrome C oxidase (CCO), an intramitochondrial component of the electron transport chain (Fig. 3) [15, 16]. CCO is a protein complex containing two copper and heme-iron groups that play a key role in the electron transport within the mitochondrial. LED-RL photostimulation of CCO directly influences reactive oxygen species (ROS) and adenosine triphosphate (ATP) production and results in increased ROS and ATP levels (Fig. 3).

Red light has also been shown to modulate a number of other mitochondrial functions, including increased intramitochondrial calcium concentration, and alterations in mitochondrial membrane potential, which may also play a role in mediating downstream effects (Fig. 3) [16]. Furthermore, while there is substantial data demonstrating the effects of redox mechanisms on cellular health and function, there is a paucity of data on how specific mitochondrial measures, such as calcium concentration, might lead to downstream cellular effects. The absence of precise mechanistic links between these other mitochondrial alterations has led many researchers to focus on studying the downstream cellular effects of LED-RL and investigating the role of visible light-associated alterations in ROS levels.

### ROS-Related Intracellular Signaling and Transcriptional Changes

For instance, altering ROS levels can release TGF-beta 1 and TGF-beta 3 from their associated latency binding proteins (Fig. 3). The interaction of these cytokines with their associated receptors is critical in the pathogenesis or prevention of skin fibrosis. Altering the levels of TGF-beta 1 versus TGF-Beta 3 bound to the TGF-beta receptor complex modulates the pro-fibrotic cascade that leads to downstream activation of key signaling molecules called SMADs and numerous growth factors that ultimately result in fibroblast proliferation and collagen biosynthesis [17, 18, 21, 22].

It is believed that visible light-associated increases in ROS levels within the cell trigger redox-sensitive transcription factors such as AP-1, NF-kB, p53, and hypoxia inducible factor 1 (HIF-1) [30]. Cellular redox changes also modulate insulin-like growth factors (IGFs), Akt/PKB, and phosphoinositide 3-kinase (PI3K) pathways, and activate mammalian target of rapamycin (mTOR) [31]. ROS-initiated alterations in these pathways often contribute to the downstream effects on transcription, cellular proliferation, migration speed, and extracellular matrix production. This suggests that ROS may be the mechanistic link between the mitochondrial effects of LED-RL and the resulting downstream transcriptional and cellular effects.

In additional to these canonical transcriptional alterations, some researchers believe that alterations in microRNA levels also play a role in LED-RL photobiomodulation (Fig. 3). However, there is currently a paucity of data investigating the specific effects of LED-RL on microRNA levels. Interestingly, research has demonstrated that laser-generated visible red light leads to specific alterations in microRNA that are associated with skin fibrosis, including microRNA-7a, 21, 29, 133b, and 192 [31, 32]. Further research is warranted to investigate the role these microRNA play in causing LED-RL-associated downstream cellular effects.

## Effects of Red Light on Cellular Functions Related to Fibrosis

### Cellular Proliferation

Modulation of mitochondrial, intracellular, and nuclear processes ultimately alter downstream cellular processes involved in skin fibroblast proliferation. For instance, fibroblast proliferation is a key contributor to the initiation and maintenance of skin fibrosis, and control of fibroblast proliferation is a critical therapeutic strategy for addressing skin fibrosis [33]. Our group has found that LED-RL is capable of inhibiting fibroblast proliferation in a dose-dependent manner [34]. Furthermore, red light does not appear to affect fibroblast viability, with no increases in apoptosis or necrosis observed [34, 35••]. This suggests that visible red light is likely modulating fibroblast function through means other than direct cellular cytotoxicity, such as through modulation of the cell cycle or autophagy.

It is likely that these alterations in proliferation are a result of alterations in the redox state of fibroblasts treated with LED-RL. While mild elevations in free radicals have been shown to increase proliferation, we have found that the dose-dependent decreases in fibroblast proliferation are associated with a dose-dependent sustained increase in ROS [35••, 36]. Therefore, it is likely that the sustained alterations in the redox state of fibroblasts treated with LED-RL and the subsequent redox-initiated alterations in the TGF-beta pathway and related pathways are contributing to the dose-dependent decrease in fibroblast proliferation.

### Cellular Migration

Furthermore, some believe that cellular migration speed may play a role in the recruitment of fibroblasts to sites of increased collagen production [3, 4]. This finding is supported by the fact that fibroblasts derived from skin affected by skin fibrosis demonstrate increased motility when compared to fibroblasts derived from normal healthy skin [37, 38]. Few studies have sought to address this potential therapeutic avenue and so the clinical effect of decreasing fibroblast motility is still unclear.

Researchers have demonstrated that the PI3K/Akt and MAPK/ERK pathways play crucial roles in the regulation of fibroblast migration, and that visible light is capable of directly activating or inhibiting the phosphorylation state of these key cell signaling molecules [32, 39–44]. Our group recently found that LED-RL increased ROS levels and decreased fibroblast migration speed in a dose-dependent manner (Fig. 3) [35••]. Additionally, we found that LED-RL also altered phospho-Akt levels (unpublished data by Jagdeo Lab). Furthermore, migration speed returned to control levels when ROS increases were blocked by the pretreatment of fibroblasts with the antioxidant resveratrol or when cells were pretreated with the PI3K/Akt inhibitor LY294002 [35••]. This suggests that LED-RL’s effects on migration may be largely mediated by increased ROS that lead to modulation of phospho-Akt levels and subsequent alterations in fibroblast migration speed. Further research is needed to investigate the role cellular migration speed plays in the pathogenesis of skin fibrosis and the clinical effects of therapeutically targeting fibroblast motility.

### Collagen Production

Fundamentally, the pathogenesis of all forms of skin fibrosis involves an increased deposition of skin collagen [33]. Therefore, suppression of collagen production is a fundamental component of any effective anti-fibrotic therapy [33]. Several studies support that visible red light is capable of modulating collagen production in vitro. Our group has demonstrated that LED-RL is capable of suppressing collagen production in human skin fibroblast cultures. In this study, fibroblasts were treated with LED-RL, and then collagen was measured using the collagen stain, picrosirius red (unpublished data by Jagdeo Lab). LED-RL resulted in decreased collagen production in a dose-dependent manner (Fig. 3). Furthermore, procollagen 1A1 levels were found to be decreased following LED-RL treatment, suggesting that this decrease in collagen levels may be due in large part to decreases in collagen subunits.

Another study investigated the effect of visible red light generated by a diode laser on murine NIH/3T3 fibroblasts [45]. They found that red light treatment inhibited TGF-beta induced fibroblast to myofibroblast differentiation and decreased collagen 1 expression. Furthermore, they found that red light was capable of upregulating matrix metalloproteinases (MMP)-2 and MMP-9, while downregulating tissue inhibitor of metalloproteinase (TIMP)-1 and TIMP-2 [45]. This suggests that red light may not only decrease collagen production, but may also change overall extracellular matrix remodeling profile. Further studies are needed evaluating the effects of red light phototherapy on in vivo collagen content and homeostasis; however, these early in vitro findings are promising.

## Limitations and Future Directions

However, red light phototherapy does possess several limitations. First, the current understanding of the biochemical mechanisms underlying visible light photobiomodulation is limited. More laboratory research is needed to characterize the key pathways involved in initiating the downstream cellular effects observed. Another limitation of the field of visible light phototherapy is that many in vitro studies are done on cultured skin fibroblasts. Fibroblast monocultures do not completely recapitulate the complex fibroblast phenotype or the extracellular milieu that contributes to skin fibrosis pathology. Thus, randomized clinical trials are needed to demonstrate visible red light’s effect on skin fibrosis.

Perhaps, one of the most critical challenges facing visible light phototherapy is the selection of appropriate dosimetry. Visible light does not have sufficient measures for evaluating the pharmacokinetics of light or its effect on in vivo tissue. Therefore, many dosing protocols are based upon observed effects. However, the fluence delivered depends on the duration of treatment, the power density of the light source, and the distance of the source from its target tissue. Differing any one variable can at times lead to different photobiomodulatory effects. For instance, while red light at fluences above 320 J/cm2 inhibit fibroblast proliferation, red light at fluences below 50 J/cm2 often promote fibroblast proliferation. Therefore, establishing standardized dosing ranges and thresholds for future basic science and clinical research studies may improve the comparability of different clinical studies.

We believe the use of commercially available LEDs as a visible light source is an exciting avenue of future research. LED-RL devices are safe, economic, and portable, and we believe are the optimal devices for future research and clinical use of visible red light.

## Conclusions

Visible light phototherapy is an emerging therapeutic modality for treatment of skin fibrosis. There is a growing body of evidence demonstrating that visible red light is capable of modulating key cellular characteristic associated with skin fibrosis. We believe that further laboratory research may elucidate the underlying mechanisms and effects involved in visible light photobiomodulation. LED-based devices are the optimal devices for red visible light phototherapy. There is a current lack of clinical trials investigating the efficacy of LED-RL to treat skin fibrosis. Randomized clinical trials are needed to demonstrate visible red light’s clinical efficacy on different types of skin fibrosis.
